# Supplementary material for: mTORC1 controls Golgi architecture and vesicle secretion by phosphorylation of SCYL1
Source: Nat Commun. 2022 Aug 10;13:4685. doi: 10.1038/s41467-022-32487-7 (PMC9365812; doi:10.1038/s41467-022-32487-7)
Supplement: Supplementary file 3 — Description of Additional Supplementary Files [file 41467_2022_32487_MOESM3_ESM.pdf]

Description of Additional Supplementary Files

Supplementary Data 1

Supplementary Table 1a: List of identified proteins comparing SILAC labeled SCYL1 KO to WT MCF7 cells (n=2 biological replicates, Exp1 and Exp2). Data are presented in Figure 2d. Protein and peptide IDs were filtered on a FDR<0.01 and minimum 1 unique peptide for protein identification. Minimum ratio count for quantification was 2. PEP: posterior error probability.
Supplementary Table 1b: Complete list of the GO term analysis presented in Figure 2e. Proteins significantly upregulated in at least one replicate of SCYL1 KO proteome over WT (outlier test, significance A, p<0.05, BH corrected) were submitted for GO term enrichment using STRING DB data analysis tool using the human genome as background. Non-redundant significant results are presented for enriched GO terms. False Decovery Rate (FDR) <0.05 (p value, BH corrected).
Supplementary Table 1c: Complete list of the GO term analysis presented in Figure 2e. Proteins significantly downregulated in at least one replicate of SCYL1 KO proteome over WT (outlier test, significance A, p<0.05, BH corrected) were submitted for GO term enrichment using STRING DB data analysis tool using the human genome as background. Non-redundant significant results are presented for enriched GO terms. False Decovery Rate (FDR) <0.05 (p value, BH corrected).

Supplementary Data 2

Supplementary Table 2a: List of identified proteins in conditioned medium (CM) comparing SILAC labeled SCYL1 KO to WT MCF7 cells. SILAC labeled SCYL1 KO clones (3A1, 1B1) were compared to WT MCF7 cells (WT). n=8 biological replicates for clone 3A1 (Exp1-Exp8) and n=2 biological replicates for clone 1B1 (Exp1-2) were performed. Data are presented in Figure 3a. Protein IDs and peptide-spectrum-matches were filtered on a FDR<0.01 and minimum 1 unique peptide for protein identification. Minimum ratio count for quantification was 2.
Supplementary Table 2b: List of EV characteristic proteins enriched in CMs of both SCYL1 KO #1 and #2 MCF7 clones compared with WT cell CM, from table ST3A. The 5 indicated categories correspond to MISEV2018 guidelines for identification of extracellular vesicles. At least one protein of categories 1a or 1b, 2a (optional 2b) and 3a or 3b must be analyzed to demonstrate the presence of EVs and their purity. Only proteins belonging to these categories and being enriched significantly in SCYL1 KO compared to WT control in at least one of the two KO clones (+) are displayed. BH: Bonferroni-Holm-corrected FDR significant test; p-value: p-value thresholded significance test A (max p-value 0.05). The intensity ratio between SCYL1 KO and WT cells is expressed as Log2-transformed value. Grey shaded area: amongst the best negative markers mentoned in MISEV2018 guidelines (category 3a), APOB was the only one identified in our results, and was negatively regulated in SCYL1 KO clones compared to WT, indicating a good purity level of the isolated EVs. In category 3b of common contaminants, only one ribosomal protein was significantly increased in SCYL1 KO clones.
Supplementary Table 2c: List of identified proteins comparing SILAC labeled SCYL1 KO and GORAB KO to WT MCF7 cells, respectively (n=2 biological replicates each, Exp1 and Exp2). Data are presented in Figure 3g. Protein IDs and peptide-spectrum-matches were filtered on a FDR<0.01 and minimum 1 unique peptide for protein identification. Minimum ratio count for quantification was 2.

Supplementary Data 3

Supplementary Table 3: *In vitro* kinase assays using FLAG-MTOR purified mTORC1 holo-complexes and HA-SCYL1 as input. Three biological replicates using ^{18}O-labelled ATP, with and without wortmanin as kinase inhibitor were performed, respectively (1-3). The three listed phosphosites were only identified in samples without wortmanin. Ser754 was manualy annotaed due to the high abundance of the respective endogneous phosphorylated peptide. Data presented in Figure 4c.

Supplementary Data 4

Supplementary Table 4a: List of identified proteins in SCYL1-BioID AP comparing SILAC labeled SCYL1-WT and mutant cells to cells expressing BirA-only. SILAC labeled HA-BirA-SCYL1 cells were compared to BirA-only cells (BirA). WT SCYL1 (SCYL1) was analyzed in 12 biological replicates (Exp01-12), mutant SCYL1 (SCYL1-S754A, _A; SCYL1-S754E, _E) were analyzed in 6 biological replicates each (Exp01-Exp06). Data are presented in Figure 7b and 7d. Protein IDs and peptide-spectrum-matches were filtered on a FDR<0.01 and minimum 1 unique peptide for protein identification. Minimum ratio count for quantification was 2.

Supplementary Table 4b: Comparative GO term enrichment analysis of SCYL1^{S754A}-BirA and SCYL1^{S754E}-BirA proximity labeling data. Proteins differentially enriched at least by a Log2 of 0.5fold between the S754A and S754E mutants (Supplementary Table ST4a) were analyzed by DAVID. p values were calculated using the human genome as background via DAVID functional annotation tool EASE Score. Data are presented in Figure 7c.
