# Supplementary material for: mTORC1 controls Golgi architecture and vesicle secretion by phosphorylation of SCYL1
Source: Nat Commun. 2022 Aug 10;13:4685. doi: 10.1038/s41467-022-32487-7 (PMC9365812; doi:10.1038/s41467-022-32487-7)
Supplement: Supplementary file 8 — Reporting Summary [file 41467_2022_32487_MOESM8_ESM.pdf]

nature portfolio

Corresponding author(s): Jörn Dengjel

Last updated by author(s): 6/16/2022

Reporting Summary

Nature Portfolio wishes to improve the reproducibility of the work that we publish. This form provides structure for consistency and transparency in reporting. For further information on Nature Portfolio policies, see our Editorial Policies and the Editorial Policy Checklist.

Statistics

For all statistical analyses, confirm that the following items are present in the figure legend, table legend, main text, or Methods section.

n/a	Confirmed	
☐	☒	The exact sample size (n) for each experimental group/condition, given as a discrete number and unit of measurement
☐	☒	A statement on whether measurements were taken from distinct samples or whether the same sample was measured repeatedly
☐	☒	The statistical test(s) used AND whether they are one- or two-sided<br>*Only common tests should be described solely by name; describe more complex techniques in the Methods section.*
☒	☐	A description of all covariates tested
☒	☐	A description of any assumptions or corrections, such as tests of normality and adjustment for multiple comparisons
☐	☒	A full description of the statistical parameters including central tendency (e.g. means) or other basic estimates (e.g. regression coefficient) AND variation (e.g. standard deviation) or associated estimates of uncertainty (e.g. confidence intervals)
☐	☒	For null hypothesis testing, the test statistic (e.g. F, t, r) with confidence intervals, effect sizes, degrees of freedom and P value noted<br>*Give P values as exact values whenever suitable.*
☒	☐	For Bayesian analysis, information on the choice of priors and Markov chain Monte Carlo settings
☒	☐	For hierarchical and complex designs, identification of the appropriate level for tests and full reporting of outcomes
☐	☒	Estimates of effect sizes (e.g. Cohen's d, Pearson's r), indicating how they were calculated

Our web collection on statistics for biologists contains articles on many of the points above.

Software and code

Policy information about availability of computer code

Data collection	Leica LAS X v.2.7.3 (Leica microsystems); Li-Cor Image Studio v.2.0.38 (Li-Cor Biosciences); Nanosight Z-NTA Software v.2.3 (Malvern Panalyticals); Volocity software suite (Perkin Elmer, Version 6.3); Xcalibur v.4.1.31.9 (Thermo Scientific)
Data analysis	Imaris v.9.7.2 and 9.8.2 (Bitplane, Oxford Instruments); Fiji v.2.3.0 (ImageJ.net); Microsoft Excel v.16.58 (Microsoft); MaxQuant (version 1.6.2.10); Perseus software v.1.5.5.3 (MaxQuant); Instant Clue v.0.10.10.20210316; Adobe Photoshop (v.22.5.1, Adobe)

For manuscripts utilizing custom algorithms or software that are central to the research but not yet described in published literature, software must be made available to editors and reviewers. We strongly encourage code deposition in a community repository (e.g. GitHub). See the Nature Portfolio guidelines for submitting code & software for further information.

Data

Policy information about availability of data

All manuscripts must include a data availability statement. This statement should provide the following information, where applicable:

- Accession codes, unique identifiers, or web links for publicly available datasets
- A description of any restrictions on data availability
- For clinical datasets or third party data, please ensure that the statement adheres to our policy

The authors declare that the data supporting the findings of this study are available within the paper and its supplementary information files, or in the source data file. Source data are provided with this paper. Raw confocal and electron microscopy images used in this study are available from the corresponding author upon request. The mass spectrometry data have been deposited to the ProteomeXchange Consortium via the PRIDE partner repository 76. (a) identifier PXD024399, username: reviewer_pxd024399@ebi.ac.uk, password: IhAmagD3, (b) identifier PXD024550, username: reviewer_pxd024550@ebi.ac.uk, password: hlipqLiC. Used public databases were: https://www.proteinatlas.org/; https://www.proteomicsdb.org/, https://www.uniprot.org/, https://www.phosphosite.org/

homeAction.action.

Field-specific reporting

Please select the one below that is the best fit for your research. If you are not sure, read the appropriate sections before making your selection.

[x] Life sciences [ ] Behavioural & social sciences [ ] Ecological, evolutionary & environmental sciences

For a reference copy of the document with all sections, see nature.com/documents/nr-reporting-summary-flat.pdf

Life sciences study design

All studies must disclose on these points even when the disclosure is negative.

Sample size	Samples size calculations were not performed as only significant changes were further considered.
Data exclusions	Data exclusions were performed for image analyses only, with the pre-defined exclusion criteria of cell death, cell division, and cell damage caused by the experimental conditions or handling.
Replication	Biological replicates indicate independent experiments; technical replicates indicate multiple repetition of each assay within the same experiment (e.g., individual microscope slides of the same condition assayed during each of the replicate experiments). Replications were successful. Data are displayed as mean (average) of at least three biological replicates, or three technical replicates of two biological replicates. Error bars represent the standard error of the mean (SEM). All differences between means were tested using standard unpaired, two-tailed Student's t-test. p-values are displayed in full in Source data and expressed with asterisks in the figures.
Randomization	Randomization was not performed as we do not face any selection bias. We study differences between predefined selected states and/or defined genotypes.
Blinding	The investigators were not blinded for these experiments as all generated data were processed in parallel using the same settings and algorithms.

Reporting for specific materials, systems and methods

We require information from authors about some types of materials, experimental systems and methods used in many studies. Here, indicate whether each material, system or method listed is relevant to your study. If you are not sure if a list item applies to your research, read the appropriate section before selecting a response.

Materials & experimental systems

n/a	Involved in the study	
[ ]	[x]	Antibodies
[ ]	[x]	Eukaryotic cell lines
[x]	[ ]	Palaeontology and archaeology
[x]	[ ]	Animals and other organisms
[x]	[ ]	Human research participants
[x]	[ ]	Clinical data
[x]	[ ]	Dual use research of concern

Methods

n/a	Involved in the study	
[x]	[ ]	ChIP-seq
[x]	[ ]	Flow cytometry
[x]	[ ]	MRI-based neuroimaging

Antibodies

Antibodies used

The rabbit polyclonal antibody specific for SCYL1 phosphorylated on serine 754 (phospho-S754-SCYL1) was raised and purified from synthetic peptide CQPRPDS(PO3H2)WGEDNW (Eurogentec). The antibody fraction recognizing the non-phosphorylated CQPRPDSWGEDNW peptide was used as anti-SCYL1 (total) in our assays.
For western blotting, the following primary antibodies were used at a 1:1000 dilution, unless otherwise stated:
mouse anti-SCYL1 (Sigma-Aldrich, SAB1402612)
mouse HRP-conjugated anti-GAPDH (Santa Cruz Biotechnologies, sc-25778HRP),
mouse anti-Phospho-p70 S6 kinase (Thr389) (Cell Signaling, 9206s),
rabbit anti- p70 S6 kinase (Cell Signaling, 9202s),
rabbit anti-p62/SQSTM1 (Cell Signaling, 5114s),
mouse anti-LC3B (Nanotools, 0231S0502),
rabbit anti-GABARAPL1 (Cell Signaling, 26632),
mouse anti-CD9 (Santa Cruz Biotechnologies, sc-13118),
mouse anti-CD81 (Santa Cruz Biotechnologies, sc-7637),
rabbit anti-calreticulin (Cell Signaling, 2891S),
rabbit anti-GORAB (Atlas antibodies, HPA027250),
mouse anti-COPB (Santa Cruz Biotechnologies, sc-393615),

mouse anti-COPA (Santa Cruz Biotechnologies, sc-398099),
rabbit anti-COPG1 (Atlas antibodies, HPA037866),
mouse anti-CHMP4A/B (Santa Cruz Biotechnologies, sc-514869),
mouse anti-CHMP5 (Santa Cruz Biotechnologies, sc-374338),
mouse anti-TSG101 (Santa Cruz Biotechnologies, sc-136111),
mouse anti-EEA1 (Santa Cruz Biotechnologies, sc-137130),
mouse anti-LAMP2 (Santa Cruz Biotechnologies, sc-18822),
mouse anti-RAB7 (abcam, ab50533),
mouse anti-LAMP2 (Santa Cruz Biotechnologies, sc-18822),
mouse HRP conjugated anti-alpha-actin (Santa Cruz Biotechnologies, sc-47778HRP).
HRP-conjugated goat anti-Rabbit IgG (Jackson ImmunoResearch, 111-035-045) 1:10'000 dilution,
HRP-conjugated goat anti-mouse IgG (Jackson ImmunoResearch, 115-035-062) 1:5'000 dilution,
anti-mouse IgG "VeriBlot" for IP secondary antibody, HRP-conjugated (ABcam, ab131368) 1:10'000 dilution,
light chain specific, HRP-conjugated AffiniPure goat anti-mouse IgG (Jackson ImmunoResearch, 115-035-174) 1:5'000 dilution,
light chain specific, HRP-IgG fraction monoclonal mouse anti-rabbit (Jackson ImmunoResearch, 211-032-171),1:10'000 dilution.
Primary antibodies were used in immunostainings at 1:100 dilution, unless otherwise stated:
rabbit anti-SCYL1 (Atlas antibodies, HPA015015),
mouse anti-GOLGIN-97/GOLGA1 (ThermoScientific, A21270),
mouse anti-LAMP2 (Santa Cruz Biotechnologies, sc-18822),
rabbit anti-mTOR (Cell Signaling, 2983S),
mouse anti-SNX1 (BD Biosciences, 611482),
mouse anti-COPA (Santa Cruz Biotechnologies, sc-398099),
rabbit anti-COPG1 (Atlas antibodies, HPA037866),
rabbit anti-TGN46 (Atlas antibodies, HPA012609; Biorad, AHP500GT),
mouse anti-GM130 (BD biosciences, #610823),
mouse anti-RAB5 (Cell Signaling, 46449),
mouse anti-RAB7 (abcam, ab137029),
rabbit anti-GORAB (Atlas antibodies, HPA027250).
Secondary antibodies for immunostainings were used at 1:2'000 dilution:
Alexa Fluor 568-conjugated anti-rabbit IgG (H+L) (ThermoScientific, A11011),
Alexa Fluor 488-conjugated anti-rabbit IgG (H+L) (ThermoScientific, A21206),
Alexa Fluor 633-conjugated anti-mouse IgG (H+L) (ThermoScientific, A21050),
Alexa Fluor 488-conjugated anti-mouse IgG (H+L) (ThermoScientific, A21202),
Alexa Fluor 633-conjugated anti-rabbit IgG (H+L) (ThermoScientific, A21071),
DyLight 405-conjugated anti-mouse IgG1 (Jackson Immunoresearch, 115-475-205) and Cy3-conjugated anti-Mouse IgG2b (Jackson Immunoresearch, 115-165-207).

Validation

All commercial primary antibodies were validated by the manufacturer's standards for human cells and applications used in this study, i.e. western blot and immunofluorescence, and were used according to manufacturer's recommendations. Reactivity against human proteins is determined by testing in at least one approved application, i.e. western blot and/or immunofluorescence.
The rabbit polyclonal antibody specific for SCYL1 phosphorylated on serine 754 (phospho-S754-SCYL1) was raised and purified from synthetic peptide CQPRPDS(PO3H2)WGEDNW (Eurogentec). The antibody fraction recognizing the non-phosphorylated CQPRPDSWGEDNW peptide was used as anti-SCYL1 (total) in our assays. Antibody validation was performed by western blotting on WT and SCYL1 KO cell extracts to show SCYL1 specificity, and against overexpressed tagged HA-SCYL1 in WT and S754A/E variant forms to show specificity for WT only, but not for phosphomutants. A treatment of western blot membranes with a phosphatase mixture blocked most phospho-specific antibody recognition, depmonstrating phospho-specificity. All valifdation results for these antibodies are displayed in supplementary figure S4.

Eukaryotic cell lines

Policy information about cell lines

Cell line source(s)

MCF7, HEK293T, A549, HeLa and hTERT-RPE-1 cells are ATCC-derived strains (ATCC® HTB-22, CRL-11268, CCL-185, CCL-2, and CRL-4000, respectively).

Authentication

Cells were authenticated by genotyping using the cell line authentication service from Microsynth AG.

Mycoplasma contamination

All cell lines used in this study were regularly tested for mycoplasma contamination using the Venor GeM Classic Mycoplasma Detection Kit (Minerva biolabs, 11-1100), and were determined to be negative.

Commonly misidentified lines
(See ICLAC register)

No misidentified cell lines were used in this study.
