# Supplementary material for: mTORC1 controls Golgi architecture and vesicle secretion by phosphorylation of SCYL1
Source: Nat Commun. 2022 Aug 10;13:4685. doi: 10.1038/s41467-022-32487-7 (PMC9365812; doi:10.1038/s41467-022-32487-7)
Supplement: Supplementary file 1 — Supplementary Information [file 41467_2022_32487_MOESM1_ESM.docx]

**mTORC1 controls Golgi architecture and vesicle secretion by phosphorylation of SCYL1**

Stéphanie Kaeser-Pebernard^1^, Christine Vionnet^1^, Muriel Mari^2,3^, Devanarayanan Siva Sankar^1^, Zehan Hu^1^, Carole Roubaty^1^, Esther Martínez-Martínez^1^, Huiyuan Zhao^4^, Miguel Spuck-Calvar^5,6^, Alke Petri-Fink^5,7^, Gregor Rainer^4^, Florian Steinberg^8^, Fulvio Reggiori^2,3,9^, Jörn Dengjel^1,^*

^1^ Department of Biology, University of Fribourg, 1700 Fribourg, Switzerland

^2^ Department of Biomedical Sciences of Cells and Systems, University of Groningen, University Medical Center Groningen, 9713AV Groningen, The Netherlands

^3^ Department of Biomedicine, Aarhus University, 8000 Aarhus, Denmark

^4^ Section of Medicine, University of Fribourg, 1700 Fribourg, Switzerland

^5^ Adolf Merkle Institute, University of Fribourg, 1700 Fribourg, Switzerland

^6^ CINBIO, Universidade de Vigo, 36310 Vigo, Spain

^7^ Department of Chemistry, University of Fribourg, 1700 Fribourg, Switzerland

^8^ Center for Biological Systems Analysis, University of Freiburg, 79104 Freiburg, Germany

^9^ Aarhus Institute of Advanced Studies (AIAS), Aarhus University, 8000 Aarhus, Denmark

**Supplementary Figures**

*Each Supplementary Figure x is attached to main Figure x (e.g. Supplementary Figure 1 is attached to Figure 1)…*

**Supplementary Figure 1:** SCYL1 delocalization to endosomal vesicles upon mTORC1 inhibition occurs in various cell types.

**Supplementary Figure 2:** Loss of *SCYL1* leads to enlarged and disorganized Golgi with high vesiculation

**Supplementary Figure 3:** *SCYL1* KO cells secrete small EVs in a *GORAB*-independent manner.

**Supplementary Figure 4:** Generation of a phospho-specific antibody against SCYL1 phosphoSer754.

**Supplementary Figure 5:** HA-SCYL1(WT) and HA-SCYL1(S754E), but not HA-SCYL1(S754A), rescue the KO phenotype.

**Supplementary Figure 1**

**Supplementary Figure 1: SCYL1 delocalization to endosomal vesicles upon mTORC1 inhibition occurs in various cell types. (a) Representative fluorescence microscopy images from MCF7, A549 and HeLa cell lines.** Widefield images from experiment displayed in Figure 1a. Cells in normal (DMEM) or treated conditions (100 nM Rapamycin 3 h or starvation by HBSS 3 h) were immunostained for endogenous SCYL1 (green). Merged pictures also display DNA (blue) stained by Hoechst 33342 reagent. **(b) Average plot profiles of SCYL1 distance from nuclei in MCF7 cells displayed in (a).** Plot profiles of SCYL1 fluorescence intensity from nuclear edge towards cell surface was measured in MCF7 cells from (a) and normalized to the maximal intensity observed on each plot. Lines represent averages of n=10 cells. Green lines= DMEM, red lines= Rapamyin 3 h. The standard deviation (SD) range between minimal and maximal values is represented by shaded areas of the same color. **(c-d) SCYL1-SNX1 co-localization events increase upon mTORC1 inhibition in RPE-1 cells.** RPE-1 cells were treated with rapamycin or vehicle for 3h, followed by fixation and staining with the indicated antibodies. The co-localization (c) between SCYL1 and TGN marker TGN46/TGOLN2 or Retromer/endosome marker SNX1 was analyzed across 10 images from two independent experiments (n=20; two-sided t-test, unequal variance). Individual data points are indicated. Error bars indicate 95% confidence interval. **(e-g) SCYL1-SNX1 co-localization events increase upon mTORC1 inhibition in MCF7 cells.** (e), Representative fluorescence microscopy pictures from WT MCF7 cells in untreated (DMEM) and treated (3 h 100 nM Rapamycin) conditions. Cells were immunostained with antibodies against SCYL1 and SNX1, and DNA was stained with Hoechst reagent. Single slices from equivalent positions within cells are shown. Yellow lines: line drawings used to generate plot profiles in (g). One representative experiment out of two biological replicates is shown. Scale bar = 10 μm. (f), thresholded Mander’s M1 Correlation coefficient showing the proportion of SNX1 colocalizing with SCYL1 in MCF7 cells, in untreated (DMEM) and treated (3 h 100 nM Rapamycin) conditions as shown in (e). n=41 (DMEM) and n=46 (Rapa) cells measured throughout two biological replicates. Measures were performed in 3D on stacks of 25 slices on average, on automatically-generated regions of interest (ROIs) defined by regions positive for SCYL1 signal. Individual data points as well as box plots are shown; center lines within the box plots indicate median values; box limits indicate the upper and lower quartiles of the plotted values, whereas upper and lower whiskers indicate the largest and smallest values when in the range of the 1.5x interquartile range (IQR) from the box limits. (g), plot profiles of (e), representing the percentage of maximal intensity in function of the distance to nuclear edge along selected plot profile lines for SCYL1 (green) and SNX1 (red). Source data for Supplementary Figure 1 are provided as Source Data file.

**Supplementary Figure 2**

**Supplementary Figure 2: Loss of *SCYL1* leads to enlarged and disorganized Golgi with high vesiculation. (a) Transmission electron microscopy analysis of *SCYL1* KO cells.** *SCYL1* KO cells display a visibly enlarged, disorganized and highly vesiculated Golgi apparatus. Multiple endosomes are also visible. D, degradative compartment; E, endosome; G, Golgi apparatus; M, mitochondria; N, nucleus; PM, plasma membrane. N: Nucleus; G: Golgi apparatus; PM: Plasma membrane. Scale bar: 500nm. One set of representative pictures is shown out of n=2 individual replicates. **(b-c) mTORC1 is inhibited and autophagic activity increased in *SCYL1 KO* cells.** (b) WT and *SCYL1* KO MCF7 cells were left untreated (DMEM), treated with 100 nM Rapamycin for 3 h (Rapa), or starved in HBSS for 3 h (Starv). In duplicate samples, 2 nM concanamycin A (conA) was added to block lysosomal degradation. Cell lysates were generated and blotted against the indicated proteins. Representative western blots of 3 biological replicates are shown. (c) Quantification of (b). Protein abundances were normalized to initial level in untreated WT cells. All data were normalized to total GAPDH levels, except for phosphorylated RPS6K which was normalized to total S6K protein levels. Error bars= SEM. **(d-e) Localization of GORAB and COPI complex in *SCYL1* KO cells**. (d) GORAB localization at the trans-Golgi network (TGN) remains unchanged in *SCYL1KO* cells. (e) The COPI complex member COPG1, which localizes at the Golgi and ERGIC compartments in WT untreated cells, is widely delocalized in *SCYL1KO* cells. (d,e) Representative fluorescence microscopy images from WT and *SCYL1KO* MCF7 cells (clone KO1), immunostained for endogenous (d) GORAB or (e) COPG1 (green) and trans-Golgi network resident GOLGA1/GOLGIN97 (red) are shown, out of n=2 biological replicates. The merged picture also displays DNA (blue) stained by Hoechst 33342 reagent. Source data and uncropped western blots for supplementary figure S2 are provided as Source Data file.

**Supplementary Figure 3**

**Supplementary Figure 3: *SCYL1* KO cells secrete small EVs in a *GORAB*-independent manner. (a-b) *SCYL1* KO cells secrete small EVs.** (a) wide-field Scanning Electron Microscopy (SEM) pictures and corresponding zoom regions as displayed in Figure 3D. Scale bar: 1 μm. (b) measurement of extracellular vesicles in WT (upper panel) and *SCYL1* KO (lower panel) conditioned extracellular medium. Ultracentrifuged, concentrated EVs from equal amounts of each cell line were laid on silicon wafers, allowing for clearer pictures and precise measurement of their diameter. WT EVs are in the medium/large EV diameter range (>200 nm), whereas *SCYL1* KO EVs show a diameter <200 nm, corresponding to the small EV category. One set of representative images, out of n=2 independent replicates, is shown. Scale bar: 1 μm. **(c-e):** ***GORAB* KO cells show SCYL1 delocalization, but no increase in EV secretion.** (c-d) Loss of *GORAB* leads to SCYL1 detachment from the TGN and relocation to cytoplasmic foci. (c) Representative fluorescence microscopy images from WT and *GORAB* KO MCF7 cells untreated (DMEM) or treated for 3 h with 100 nM Rapamycin (Rapa). Cells were immunostained for endogenous SCYL1 (green) and trans-Golgi network resident marker GOLGIN-97/GOLGA1 (red). Blue channel: Hoechst reagent, marking DNA. Yellow lines: line drawings used to generate plot profiles in (d) n=2 independent replicates. (d) plot profiles of (c), representing the percentage of maximal intensity in function of the distance to nuclear edge along selected plot profile lines for SCYL1 (green) and GOLGIN-97/GOLGA1 (red) for the indicated cell lines and conditions. (e) *GORAB* KO do not phenocopy *SCYL1* KO cells for EV secretion. Extracellular protein content analysis of WT, *SCYL1* KO and *GORAB* KO MCF7 cells, in untreated (DMEM) and rapamycin-treated conditions (Rapa – 100 nM, 3 h). Whole cell lysates and ultrafiltered (UF) CM containing EVs harvested from the same cells were compared by western blotting. To normalize protein contents, equal amounts of cells were used for fractionation and EV purification. Source data and uncropped western blots for supplementary figure S3 are provided as Source Data file.

**Supplementary Figure 4**

**Supplementary Figure 4: Generation of a phospho-specific antibody against SCYL1 phosphoSer754.** **(a-b)** **The phospho-specific antibody generated against SCYL1 Ser754 is 80% specific to the phosphorylated form.** (a) western blot analysis of HA-SCYL1 WT, and its phospho-null S754A, or phospho-mimicking S754E mutants forms, with antibodies specific for total (SCYL1 total) or phosphorylated (SCYL1-P-S754). Detections of phosphorylated (P-T389) or total RPS6K protein were used as control. (b) quantification of (a); SCYL1-P-S754 antibody recognizes the phosphorylated S754 WT form with ca. 80% specificity. **(c) Phosphatase treatment reduces phospho-site specific antibody binding.** The two membranes (top/bottom) were generated and treated simultaneously. Lysates were generated and analyzed by western blot using indicated antibodies. Overnight treatment with lambda and alkaline phosphatases of the bottom membrane led to a decrease of phosphorylation-dependent signals. One representative experiment out of n=2 independent replicates is shown. Source data and uncropped western blots for supplementary figure S4 are provided as Source Data file.

**Supplementary Figure 5**

**Supplementary Figure 5:** **HA-SCYL1(WT) and HA-SCYL1(S754E), but not HA-SCYL1(S754A), rescue the KO phenotype. (a)** **Lentiviral-mediated re-expression of HA-SCYL1(WT), HA-SCYL1(S754A) and HA-SCYL1(S754E) variants in MCF7 *SCYL1* KO cells.** Transgenic expression was induced with two different doxycycline concentrations (1 or 2 μg/ml) for 24 and 48 h, and equal amounts of protein extracts were monitored by western blotting for HA-SCYL1 expression levels. Actin was used as loading control. To avoid saturation, the 1 μg/ml doxycycline concentration was chosen in all subsequent assays. Both membranes were developed simultaneously, i.e. band intensity differences between membranes can be compared. One representative experiment, out of n=3 independent replicates, is shown. **(b)** **Golgi structure depends on SCYL1 Ser754 phosphorylation.** Two additional TEM pictures of MCF7 *SCYL1* KO cells re-expressing either HA-SCYL1(WT), HA-SCYL1(S754A) or HA-SCYL1(S754E) upon 24 h doxycycline induction are shown, to complement Figure 5A. D, degradative compartment; E, endosome; G, Golgi apparatus; M, mitochondria; N, nucleus; PM, plasma membrane. N: Nucleus; G: Golgi apparatus; PM: Plasma membrane; #: lipid droplets. One set of representative pictures, out of n=2 individual replicates, is shown. Scale bar: 500 nm. Source data and uncropped western blots for supplementary figure S5 are provided as Source Data file.
